# A Randomized Study to Determine the Effect of a Culturally Focused Video Intervention on Improving HPV Vaccine Intentions in a Christian Population in the United States

**DOI:** 10.1007/s10900-024-01327-8

**Published:** 2024-02-23

**Authors:** David S. Redd, Jessica D. Altman, Jamie L. Jensen, Chantel D. Sloan-Aagard, Triston B. Crook, Aaron E. Asay, Bryce U. Nielson, Ruth J. Larson, Dashiell S. Miner, Brian D. Poole

**Affiliations:** 1 Department of Microbiology and Molecular Biology, Brigham Young University, 3138 LSB, Provo, UT 84602 USA; 2 Department of Biology, Brigham Young University, Provo, UT USA; 3 Department of Public Health, Brigham Young University, Provo, UT USA

**Keywords:** Vaccines, Vaccine Hesitancy, Vaccine Intention, Human Papillomavirus, HPV, Cultural Focus

## Abstract

Safe and effective vaccines have been developed that protect against high-risk strains of HPV, but uptake is relatively low. We previously identified factors such as sexual attitudes and HPV knowledge that impact the intent of Christian parents to vaccinate their children against HPV. We hypothesized that culturally specific interventions in the form of short videos would be effective at improving HPV vaccine intentions and attitudes. We made three short educational videos, one with a Christian focus, one informational about HPV, and one control. Videos were distributed electronically with accompanying surveys, and responses were measured before and after watching a randomly selected video. The religious-focused and educational interventions significantly (*p* < 0.0001, *p* = 0.0015) improved intentions towards HPV vaccination. The religiously-focused video also significantly diminished the belief that the HPV vaccine is unnecessary because of a family’s values (*p* = 0.014). Parents significantly credited both interventions with improving their intent to vaccinate their children against HPV (*p* < 0.001 for both). These results suggest that culturally focused educational interventions are effective at influencing vaccine intentions and attitudes, even when those are based on religious or cultural feelings. Highly specific interventions are likely to be necessary for optimal improvement in vaccine hesitancy.

**Supplementary Information:**

The online version contains supplementary material available at 10.1007/s10900-024-01327-8.

## Introduction

Vaccines have drastically reduced the incidence of once common diseases. Despite the past success of vaccination programs there has been an increase in vaccine hesitancy, coupled with a resurgence of infectious diseases [1, 2]. In addition to permitting previously controlled diseases to reemerge, vaccine hesitancy negatively impacts the effectiveness of newly developed and released vaccines. Effective vaccines have been developed against high-risk HPV subtypes. These vaccines have sharply reduced the incidence of the strains covered by the vaccine in regions with high uptake [3], as well as the incidence of cervical cancer [4, 5].

Vaccine hesitancy is a difficult problem to address. Although vaccines are a safe and effective method of preventing disease, there have been concerns about their use almost since their inception. Vaccine hesitancy has evolved over time based on misconceptions, fears, and changes in public perceptions. In 2019 the WHO listed vaccine hesitance as one of the top ten threats to global health [6]. Vaccine hesitancy is defined as a delay or refusal to vaccinate despite availability of vaccination. Vaccine acceptance is the norm in the majority of global populations, however, a smaller subset of the population delay vaccination or refuse certain vaccines [7].

A myriad of factors influence vaccine hesitancy. Lack of access to accurate information and the spread of misinformation through social media has a significant negative impact on confidence in vaccines, especially on those predisposed towards conspiratorial mindsets [8]. Misinformation can quickly spread through social media, making it difficult to differentiate between fact and fabrication [9]. In addition to the rapid spread of misinformation, changes in the medical system have greatly constrained the time health care providers have per appointment, making it difficult for them to educate and address the concerns of their patients [9, 10]. The low incidence of contagious diseases due to vaccination has led to the perception that the risk posed by these diseases is also low [11, 12]. Due to the success of vaccination in preventing most major disease outbreaks, fear has shifted away from vaccine-preventable diseases to fear of the vaccines themselves [13].

The Advisory Committee on Immunization Practices (ACIP) recommends that all children aged 11 to 12 receive HPV vaccination. Despite ACIP recommendations, vaccination rates remain low. In 2013 57.3% of girls and 34.6% of boys initiated an HPV vaccination series in the United States. Less than 40% of girls and less than 15% of boys completed the series [14]. Among those with health insurance, HPV vaccination ranged from 37.8 to 24.9% in 2017 [15]. HPV vaccination has improved since then, reaching 61.7% of adolescents in 2021, but that number is still below the recommended level of 80% [16]. These numbers are mainly based on nationwide survey data, but a study examining corroborating medical records found that HPV vaccination is often under-reported, and that the true vaccination rate may be as high as 71.5%, at least for those children with medical providers [17].

There are many barriers that could prevent parents from vaccinating their children against HPV. Some previously-identified barriers include safety concerns, lack of knowledge about HPV, financial concerns, parental attitudes, lack of information about HPV vaccines, concerns about the vaccines’ effect on sexual behavior, and low perceived risk of HPV infection [18–20]. Disruptions to normally scheduled vaccinations were common due to the COVID-19 pandemic. Several measures used by the CDC indicate decreased HPV vaccination during the COVID-19 pandemic between 9 and 24% [21]. Surveys of primary care physicians also indicate that the pandemic lead to a decrease in HPV vaccination [22].

We previously identified factors that impact parental intent to vaccinate their children against HPV. We found that the more knowledge parents have about HPV and the better they understand the risks presented by infection, the higher their intent to vaccinate. We also found that parents who feel that religious adherence provides protection against HPV have lower intent to vaccinate [23]. Multiple other studies have also shown that religious and spiritual beliefs impact HPV vaccine uptake [24, 25]. We also previously found that talking to those who have experienced vaccine-preventable diseases improves vaccine attitudes [6]. This approach was reinforced by a study that found that a video intervention showing a cervical cancer survivor temporarily boosted HPV vaccine attitudes in Japan [26], although this change diminished over time. Primary care personnel believe that trustworthiness, targeted strategies, and vaccine education, among other strategies, would be effective in helping to make up for the lower HPV vaccination rates [27]. Culturally-targeted interventions have been shown to be effective in a Christian population; in one randomized control study, pro-HPV vaccination messages were configured in the form of bible stories. This had a significant impact on intent to vaccinate [28].

The objective of this study was to determine if a culturally relevant story from a cervical cancer survivor would be sufficient to improve intent to vaccinate against HPV in a Christian population. As secondary objectives, we sought to test the effectiveness of this story on altering specific beliefs that hinder HPV vaccine acceptance in this population, and to test the effectiveness of vaccine education on HPV acceptance. We designed two educational interventions in the form of short educational films. We hypothesized that a story from a cervical cancer survivor with an explicitly Christian narrative would be effective at improving attitudes towards HPV vaccination in a Christian population. We further hypothesized that a brief educational video emphasizing the benefit of HPV vaccination would effectively improve vaccine intention. We designed and filmed educational videos and tested their effectiveness using surveys.

## Materials and Methods

### Objectives

**Fig. 1:**
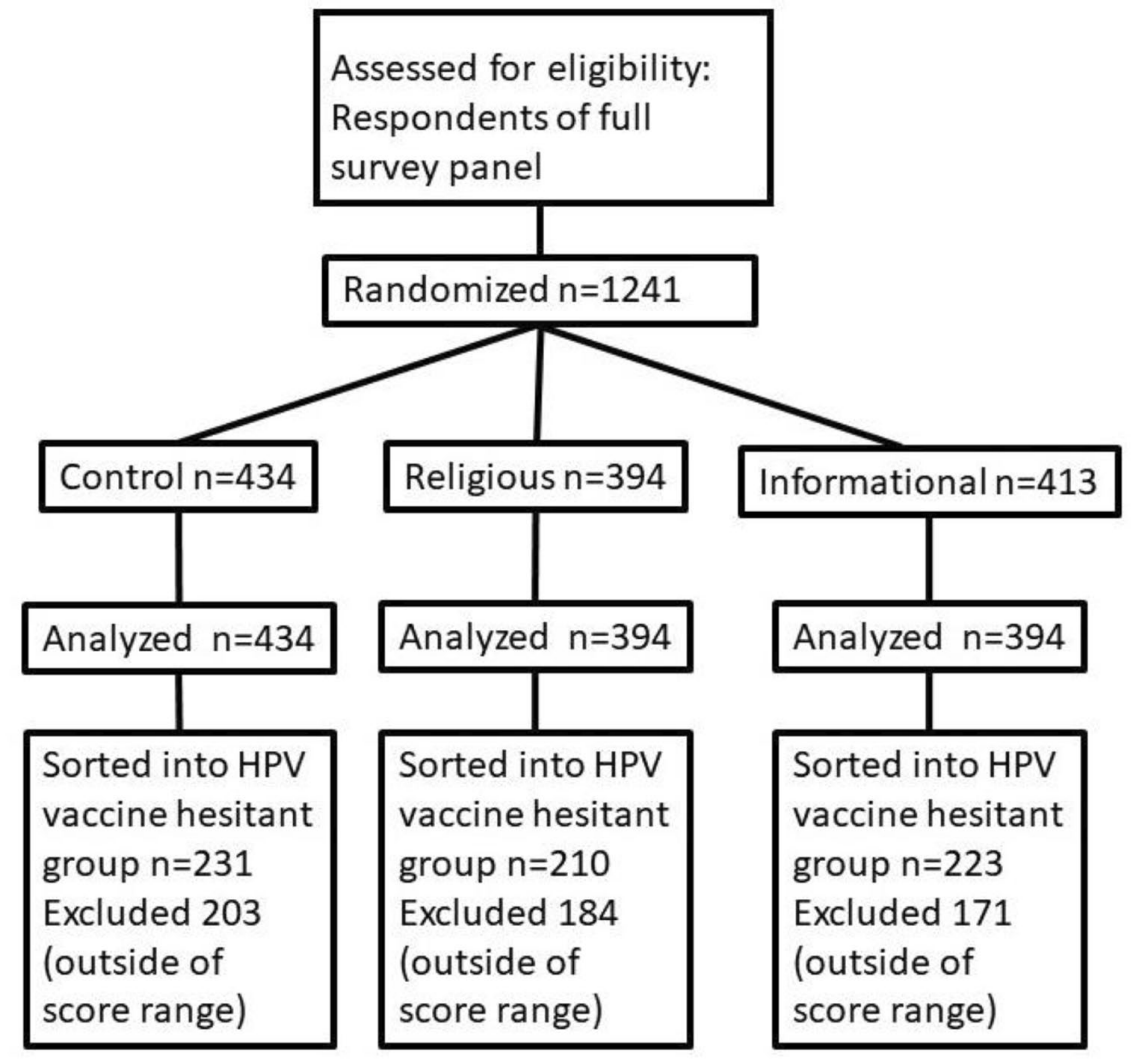
Study flow diagram. 1241 study subjects meeting the inclusion criteria (Self-identified Christian, Parent of child under 11) were recruited from professional study panels. Subjects were randomly assigned to view the Control, Religious, or Informational educational video intervention. Differences between groups were evaluated for each objective. Groups were then subdivided and the HPV vaccine hesitant members were measured against each other for each objective

We used a survey to test all objectives. The primary objective, to determine if a culturally relevant story from a cervical cancer survivor would be sufficient to improve intent to vaccinate against HPV in a Christian population, was tested using an embedded video with a story from a Christian survivor of cervical cancer. Vaccination intent was measured before and after the video and analyzed for significance using the Wilcoxon signed rank test. The same approach was used for aim 3: to test the effectiveness of vaccine education on HPV intent to vaccinate. Aim 2, to test the effectiveness of the survivor’s story on altering specific beliefs that hinder HPV vaccine acceptance in this population, was tested by comparing responses to a question about how those beliefs affect intent to vaccinate before and after the video. Significance was determined using the Wilcoxon-signed rank test for this aim as well. We attempt to follow the CONSORT guidelines in this report [29] (Supplemental Table 1) (Fig. 1).

### Survey Description

We designed a survey to examine the possible factors we believed contributed to intent to vaccinate against HPV. The survey was based on our prior work [23]. The survey was checked for face validity by a virologist (Dr. Poole), a specialist in biological education and religious influences (Dr. Jensen), and a public health expert (Dr. Sloan-Aagard). Intelligibility was checked by at least two undergraduate students. We used confirmatory factor analysis, based on the factors identified a priori by face validity, to test the measurement portion of our model and to obtain construct validity for each of the factors. CFA was performed on related subsets of factors with a request for modification indices. Items were removed until fit indices [root mean square error approximation (RMSEA), comparative fit index (CFI), Tucker-Lewis index (TLI), and standardized root mean square residual (SRMR)] were acceptable. Instruments were combined into a full measurement model to ensure fit. The following latent factors were included: Beliefs that religious adherence protects against HPV, Intent to vaccinate, Positive attitudes toward vaccines, HPV knowledge, Vaccine knowledge, Religiosity measured by three factors (Religious practice, Religious Influence, and Religious hope), Pro-vaccine religious views, Religious encouragement of premarital abstinence, Parent/peer influence on sexual behavior, and Trust in modern medicine. The few items that were removed due to lack of fit are indicated on the survey included in the Supplementary Files. CFA statistics for models are shown in Table 1.

**Table 1:** Fit Statistics for Each Measurement Model (*N* = 1,241)

Model (Latent Variables)	TLI	CFI	CFI	SRMR	Chi-square Test		
					${X}^{2}$	df	*p*-value
Model A (Religious Practice, Pro-Vaccine Religious Views, Trust in Modern Medicine, Vaccine Knowledge, Positive Attitudes Toward Vaccines, Intent to Vaccinate)	0.948	0.955	0.041	0.055	533.49	308	< 0.001
Model B (Religious Practice, Religious Encouragement of Premarital Abstinence, Beliefs that Religious Adherence Protects Against HPV, HPV Knowledge, Intent to Vaccinate)	0.927	0.938	0.058	0.065	438.50	177	< 0.001
Model for Remaining Variables (Religious Influence, Religious Hope, Parent/Peer Influence on Sexual Behavior, Fear of HPV Vaccine Side-effects)	0.966	0.973	0.041	0.045	166.61	96	< 0.001
Combined Model	0.902	0.911	0.042	0.065	2401.51	1348	< 0.001

The survey was composed of 3 parts. The first section covered demographic information and our latent variables. The second section introduced the Educational Video intervention. The final section of the survey assessed participants’ views and intentions post-intervention by repeating many of the questions contained in the first section. The entire survey is available in the supplemental information (Supplementary Table 2).

### Educational Video Intervention

We made three intervention videos. These were randomly assigned by the survey software in a 1:1:1 ratio, using the Evenly Present Elements Randomization tool from Qualtrics. In a parallel trial format, each participant received only one intervention. The first video was a control and contained information on an adenovirus, but nothing about HPV or vaccines in general. The second video will be referred to as “Religious.” It is a story told by a devout Christian cervical cancer survivor who discussed her experiences and advocated for getting vaccinated. The subject emphasized her faith and spiritual experiences along with her experience with cancer. The purpose of this video was to attempt to destigmatize HPV infection and vaccination in this community. This approach fits within a social identity theory framework [30]. We previously found an association between the perception that HPV does not affect practicing Christians, as shown by responses to the statement “I do not need to vaccinate my children against HPV because HPV is sexually transmitted, therefore my family’s values will protect my children from contracting HPV” and diminished intent to vaccinate [23]. We included this statement in the current survey to test the effects of the intervention on this belief. The study subject was told to simply tell her story and to emphasize her faith. The rationale for this video was our prior finding that hearing the story of vaccine-preventable disease survivors improves vaccine attitudes [6]. We decided to further test this finding by allowing respondents to hear the story of a cervical cancer survivor.

The third video was an educational intervention that contained scientific facts about human papillomaviruses, the diseases they cause, how they are transmitted, and how vaccination protects against them. No attempt was made to debunk wrong information, merely to present facts about HPV and present the benefits of vaccination. This video is referred to as “Informational.” The information provided was based on information presented in a course taught by Dr. Poole, which was shown to improve attitudes towards vaccination [6]. It was also based on recommendations to primary health care personnel about effective messages: trustworthiness, link to cancer, and vaccine safety [27]. The videos can be accessed here: Control: https://youtu.be/crPM3FQaRUE, Religious: https://youtu.be/B-V8ZnyqDCE, Informational :https://youtu.be/KBktpNr9RaA .

### Randomization

The survey used a 1:1:1 randomization strategy where each participant was assigned a random survey until the total desired number of participants was reached. The assignment was made by random selection by the Qualtrics software. The survey participants were blinded in that they were unaware of the existence of the video interventions that they were not selected for.

The survey was administered nationwide through the survey company Qualtrics. Subjects were included if they self-identified as Christian and had at least one child under the age of 11. Since the test run of the survey population (50 responses) showed an overabundance of highly educated individuals, quotas were instituted to select a survey population that matched national census data in terms of education. The data was cleaned by Qualtrics by examining length of time to take the survey, completion, and identifying any inappropriate runs of responses. The survey was open from April 29, 2022 to June 15, 2022. The trial was ended because the required number of participants was reached. No unexpected harms or unintended effects were seen.

The questions “I am likely to vaccinate (or have already vaccinated) my children against HPV,” “I am likely to recommend that others vaccinate their children against HPV.”, “I will (or already have) vaccinated both my daughters and sons against HPV” and “The HPV vaccine will protect my children in the case of sexual assault.” were summed for each participant and used to generate a “HPV Vaccine intention score.” This was used to determine effectiveness of the different video interventions on intent to vaccinate against HPV. This score was also used to identify participants who were deemed “HPV vaccine hesitant.” These individuals had a score between 9 and 15, where the possible range of scores was 4–20. These values were chosen since to obtain a score of 8 or less, the average choice would have been at most “Disagree” with each item, and to obtain a score of 16–20 the average choice would have been at least “Agree” with each item on the scale. Scores between 9 and 15 therefore indicate some lack of conviction. We also removed anyone who answered a “Completely vaccinated” on the question “The HPV vaccine is given in several doses. Please indicate how complete your children’s HPV vaccinations are,” in order to only examine those whose children are not already HPV vaccinated. These changes left us with 367 vaccine hesitant respondents. HPV vaccine hesitant individuals were analyzed independently using the same tests as for the entire population.

The question “I do not need to vaccinate my children against HPV because HPV is sexually transmitted, therefore my family’s values will protect my children from contracting HPV.” was previously found to be significantly negatively associated with intent to vaccinate. We repeated this question in this survey to determine if the culturally-specific story or the informational message would be able to change this perception.

Overall vaccine attitudes were also examined before and after each video using the items “Vaccines are more helpful than harmful,” “Vaccines often have severe side effects,” “Vaccines contain dangerous toxins” and “Vaccines are effective at preventing disease.”

### Ethical Approval

The study was approved by the Institutional Review Board of Brigham Young University, protocol number IRB2022-194. Informed consent was obtained electronically from all participants before they began the survey.

### Statistical Analysis

Power analysis using a 5-point Likert Scale with a standard deviation of 0.75, with a meaningful difference of 0.3, an alpha of 0.05 and a beta of 0.2, indicated that we would need 100 HPV-vaccine hesitant participants per group, or 300 HPV vaccine-hesitant participants in all, to determine effectiveness of the interventions. Based on prior results where 1 in 4 respondents was vaccine-hesitant [6], we had a goal to recruit 1200 survey participants. In actuality, 1241 were recruited, which included 612 HPV-vaccine hesitant individuals, exceeding the required minimum sample size.

Change in responses between items pre-and post intervention were compared using the Wilcoxon signed-rank test. Self-identified effectiveness of the different videos (Please indicate how much you agree with the following statement: After watching the video, I am more likely to vaccinate my children against HPV) and post-intervention intent scores between different videos were evaluated using the Mann-Whitney U Test. Alluvial plots were generated using R. Correlation between answers to each individual quantitative question and HPV vaccination intention scores indicated in “survey description” was done using Pearson correlation. Chi-square analysis was performed to examine the effects of qualitative variables on HPV attitudes. Effect size was calculated using Cohen’s *d* score. We used nonparametric tests because most of the questions used an ordinal scale and there was no guarantee of normal distribution, therefore nonparametric tests were warranted.

## Results

The survey was administered across the United States and received 1241 responses. Demographics of the respondents are shown in (Table 2). Most (83.8%) of our survey participants were between 25 and 45 years old. This age range is likely because one of our selection criteria was parents with children younger than 11. The population was predominantly white. The average number of children per survey participant was two. The majority (65%) of our sample population was female. The majority (62.3%) of participants were married. This is somewhat higher than the national average but not unusual based on our selection criteria. Less than half (57.86%) of our population had completed a college degree. The national median household income is ~$67,000, so our sample income was also fairly representative of the national average ($70,784).

**Table 2:** Demographics of the survey population. The survey was distributed nationwide using professional survey panels. Filters were applied for Christian religion, children under 11, and education corresponding to national rates. As expected, most of our population was married. Household income was in line with expected values. *N* = 1241

Category	Number	Percent of Total Responses
Age (*N* = 1,240)		
Less than 18 18–25 26–35 36–45 46–55 Over 55	0 25 414 626 125 50	0% 2.02% 33.39% 50.48% 10.08% 4.03%
Number of children (*N* = 1,240)		
1 2 More than 2	318 523 399	25.65% 42.18% 32.18%
Race/Ethnicity (*N* = 1,240)		
American Indian or Alaskan Native Asian Black or African American Hispanic or Latino Native Hawaiian or Pacific Islander White Prefer not to answer Other	19 13 157 103 6 930 4 8	1.53% 1.05% 12.66% 8.31% 0.48% 75.00% 0.32% 0.65%
Gender/Sex (*N* = 1,240)		
Male Female Non-binary/third gender	435 804 1	35.08% 64.84% 0.08%
Marital Status (*N* = 1,237)		
Single Partnered Married Divorced Widow/widower	204 126 784 99 24	16.49% 10.19% 63.38% 8.00% 1.94%
Education (*N* = 1,240)		
Have not finished high school Finished high school Some college Associate’s degree Bachelor’s degree Post-baccalaureate/professional degree	54 369 224 132 212 249	4.35% 29.76% 18.06% 10.65% 17.10% 20.08%
Household Income (*N* = 1,240)		
Less than $5,000 $5,000-$9,999 $10,000-$14,999 $15,000-$19,999 $20,000-$29,999 $30,000-$39,999 $40,000-$49,999 $50,000-$59,999 $60,000-$74,999 $75,000-$99,999 $100,000-$124,000 $125,000-$149,999 $150,000 or more	54 35 35 52 134 116 93 103 98 151 124 133 112	4.35% 2.82% 2.82% 4.19% 10.81% 9.35% 7.50% 8.31% 7.90% 12.18% 10.00% 10.73% 9.03%

**Fig. 2:**
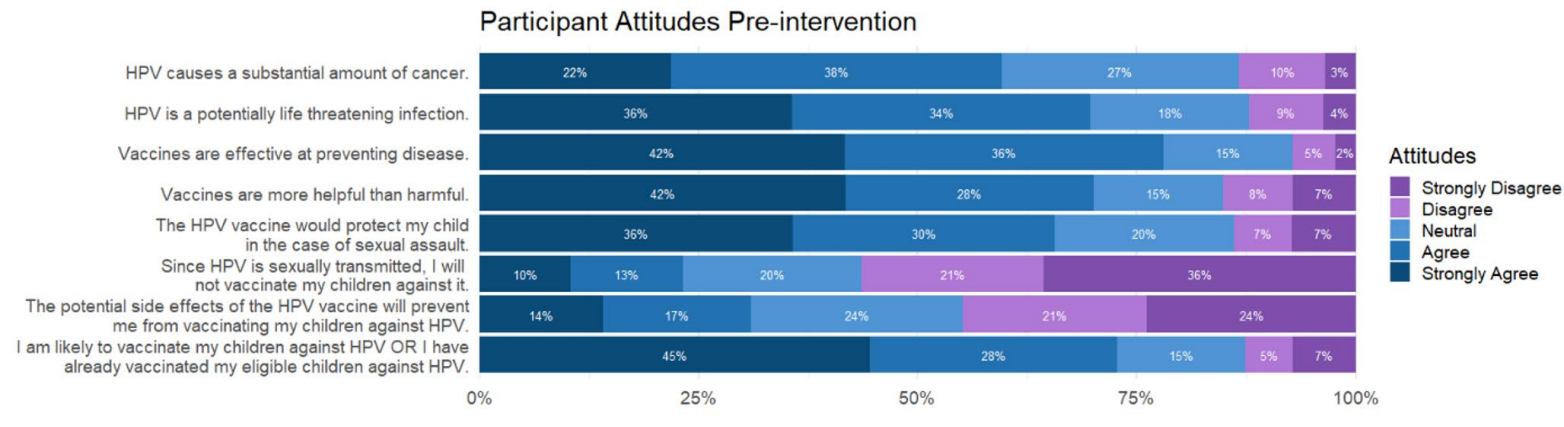
Descriptive survey results. Participants were asked a series of questions pre-intervention to determine baseline intentions toward vaccines and HPV. More than a majority of the survey respondents viewed HPV as a substantial cause of cancer and a potentially life threatening infection. Most of the respondents (78%) believed that vaccines were effective, and 70% believed they were more helpful than harmful. A small but important population (23%) would not vaccinate their children because of the sexually transmitted nature of HPV infection

Half of the participants in our population viewed HPV as a major cause of cancer. The majority of the sample (70%) viewed vaccines favorably. Most of the participants (73%) indicated that they were likely to vaccinate their children against HPV. Of the survey population, 23% (10% strongly agreed, 13% somewhat agreed) indicated that they would not vaccinate their children against HPV because of the sexually transmitted nature of the infection (Fig. 2).

**Fig. 3:**
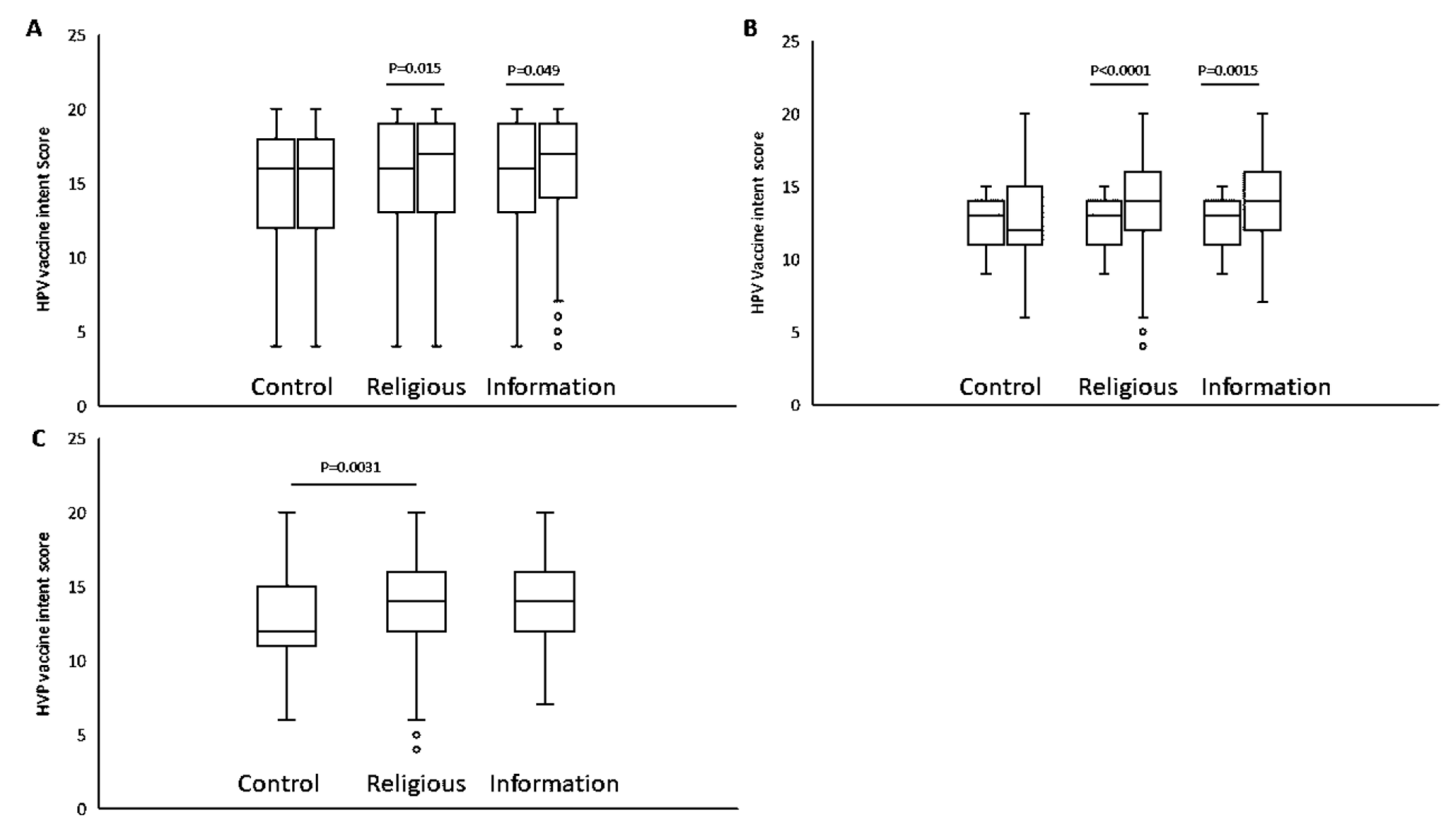
Change in HPV vaccine intention after video intervention. The questions pertaining to HPV vaccine intention were asked in the pre-video portion of the survey, and again after the video intervention. Responses to each question were summed to produce a “HPV Vaccine Attitude Score.” (**A**) Pre-and post-responses for the entire sample were compared using the Wilcoxon Signed-rank test. The mean score significantly (*p* < 0.015, effect size 0.12,) increased after the religious video intervention. The informational video also showed significance (*p* = 0.049, effect size 0.14). The control intervention was not significant. Control *n* = 433, religious *n* = 394, informational *n* = 412. (**B**) Respondents were stratified according to their initial HPV vaccine intention score. Those deemed HPV vaccine hesitant were analyzed for changes in their intention score. The religious video had a highly significant effect (*p* < 0.0001, effect size 0.47). The informational video also had a significant effect (*p* = 0.0015, effect size 0.41). The control video did not have a significant effect in this population (*p* = 0.71, effect size 0.0913) (C) Post-video vaccine intention scores were compared using the Mann-Whitney U test in the vaccine hesitant population. The religious video showed significantly higher vaccine intention than the control (*p* = 0.0031). The informational video was not significantly different than the control (*p* = 0.77). *N* = 366 total. Control 123; Religious 131; Informational 114)

A summary of the outcomes for each objective can be found in (Table 3). We were able to compare 434 respondents who watched the control video, 394 who watched the religious-focused video, and 413 who watched the informational video. Participants for each video were randomly assigned. We do not know why the groups had slightly unequal numbers. It may relate to survey participants dropping out since the religious-focused video was longer than the other two. We found that the religious-focused video significantly (*p* < 0.015, effect size 0.12) increased the HPV vaccine intention score when compared to the score in the pre-video survey. The informational video also led to a barely significant change in responses to this question (*p* = 0.049, effect size 0.14), but the control video had no significant effect (*p* = 0.79) (Fig. 3).

**Table 3:** Outcomes of the study objectives

Objective	Distribution					Mean	Median	Mode	Range	St. Dev.
**Objective 1: Change in Intent to vaccinate after religious video**	**Anti-HPV vaccine (4–8)**	**HPV vaccine hesitant (9–15)**	**Pro-HPV vaccine (16–20)**							
Total Population Pre-video (*N* = 394)	7.1% (28)	37.3% (147)	55.6% (219)			15.3	16	20	4–20	4.1
Total population Post-Video (*N* = 394)	7.1% (28)	30.7% (121)	62.2% (245)			15.8	17	20	4–20	4.1
HPV vaccine hesitant Pre-video (*N* = 222)	0%	100% (222)	0%			12.6	13	12	9–15	1.8
HPV vaccine hesitant post-video(*N* = 222)	6.9% (15)	64.9% (144)	28.2% (63)			13.8	14	12	4–20	3.2
**Objective 2: Change in belief that Culture protects against HPV**	**1 Strongly Disagree**	**2** **Somewhat Disagree**	**3** **Neither Agree Nor Disagree**	**4** **Somewhat Agree**	**5** **Strongly Agree**					
Total population Pre-Video (*N* = 394)	39.8% (157)	20.8% (82)	18.5% (73)	9.6% (38)	11.1% (44)	2.3	2	1	1–5	1.4
Total Population Post-Video (*N* = 394)	49.5% (195)	17.0% (67)	14.5% (57)	8.6% (34)	10.4% (41)	2.1	2	1	1–5	1.4
HPV vaccine hesitant Pre-Video (*N* = 222)	19.1% (43)	26.7% (59)	36.6% (81)	9.2% (20)	8.4% (19)	2.6	3	3	1–5	1.1
HPV vaccine hesitant Post-Video (*N* = 222)	34.4% (76)	23.7% (53)	28.2% (62)	8.4% (19)	5.3% (12)	2.3	2	1	1–5	1.2
**Objective 3: Change in intent to vaccinate after information video**	**Anti-HPV vaccine (4–8)**	**HPV vaccine hesitant (9–15)**	**Pro-HPV vaccine (16–20)**							
Total population pre-video (*N* = 413)	9.0% (37)	33.0% (136)	58.0% (240)			15.3	16	20	4–20	4.2
Total Population Post-video (*N* = 413)	6.8% (28)	31.1% (128)	62.1% (257)			15.9	17	20	4–20	3.9
HPV vaccine hesitant Pre-video (*N* = 210)	0%	100% (210)	0%			12.7	13	15	9–15	1.8
HPV vaccine hesitant Post-video (*N* = 210)	6.1% (13)	67.8% (142)	26.1% (55)			13.7	14	12	7–20	3.0

Although the videos had a significant effect on intent to vaccinate, the effect scores were low. This is likely due to the fact that a majority of the survey population was already committed to vaccination or strongly anti-vaccination, so the videos did not have a large effect on these groups. To test the effect on those who were not as committed, we examined the change in attitudes after watching the videos for those who scored between 9 and 15 (inclusive) on the initial HPV vaccine intention score (possible scores are from 4 to 20, with 4 items included), and who indicated that their children were not fully vaccinated. These respondents were designated “HPV vaccine hesitant.” For this population, the religious video had a highly significant effect, and the information video showed a moderately significant change. The control video was not significant (*p* = 0.71). The religious video was highly significant (*p* < 0.0001) as was the informational video (*p* = 0.0015). The effect sizes were also substantially higher for the religious (0.47) and the informational (0.41) videos than the control (0.09).

The post-video vaccine intention scores were also compared between videos for the vaccine-hesitant population. Those who watched the religious video had significantly higher post-vaccine intentions than those who watched the control video (*p* = 0.0015). The information video did not show a significant difference from the control video (*p* = 0.77).

**Fig. 4:**
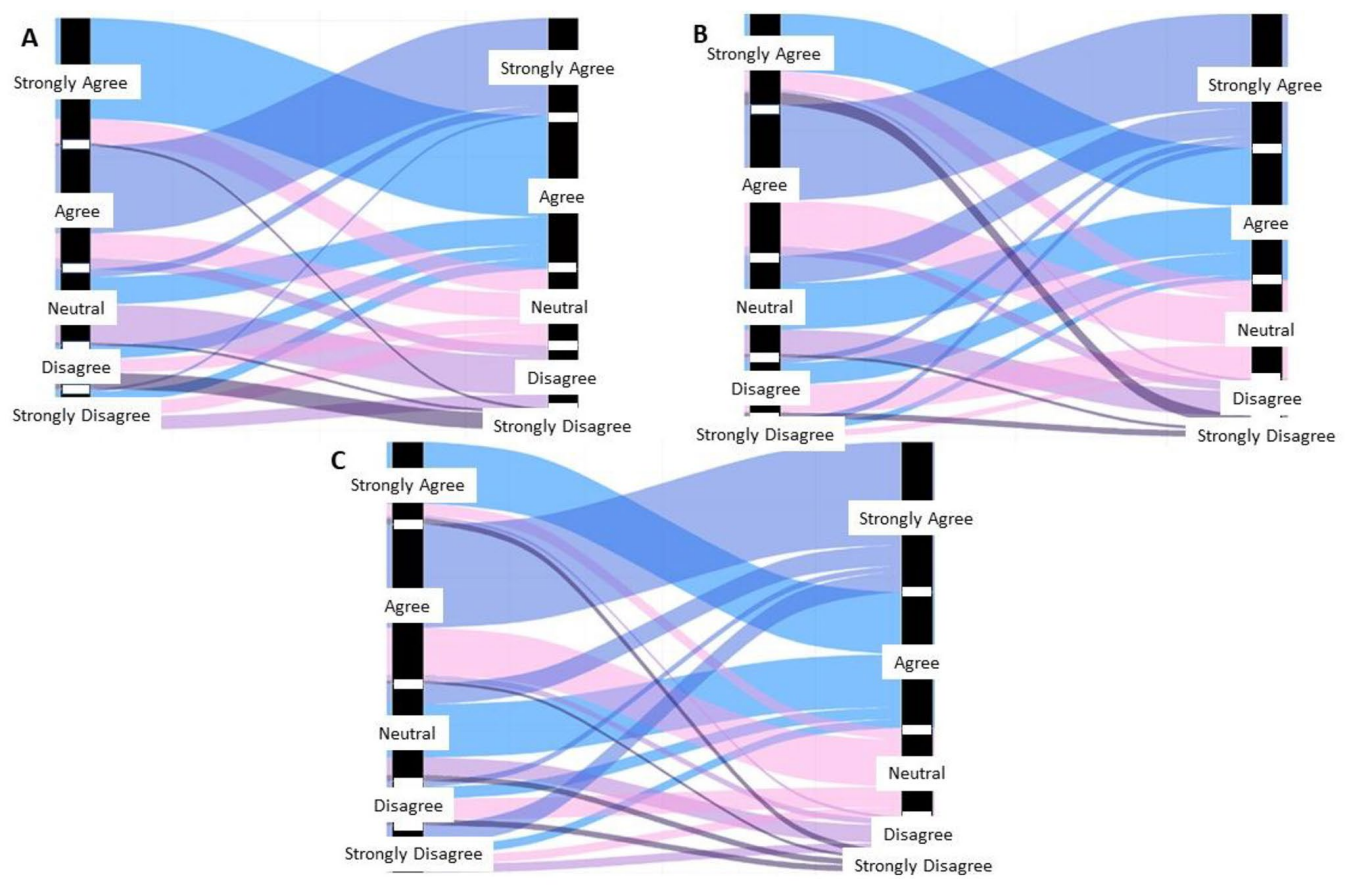
Change in HPV vaccination intent based on video intervention. Survey participants were asked to indicate how the felt about the statement “I am likely to vaccinate my children against HPV OR I have already vaccinated my eligible children against HPV.” before and after viewing the intervention video. Data for participants who indicated no change in attitude were removed for this figure. The colors correspond to participant attitudes post intervention. (**A**) Control intervention. (**B**) Religious-focused intervention. (**C**) Informational intervention

Alluvial analysis was performed to examine the changes in intent to vaccinate against HPV after the video intervention (Fig. 4**).** The religious intervention had a positive effect, especially on strengthening the intent to vaccinate of those who already agreed with the statement. The intervention also increased intent to vaccinate in a portion of participants who were neutral, disagreed, or strongly disagreed before with an intent to vaccinate. The informational intervention appeared to be somewhat effective at improving the intent of participants who had neutral views before viewing the intervention. More than half of the participants who strongly disagreed with the statement before the informational intervention indicated that they agree or strongly agreed after the intervention.

**Fig. 5:**
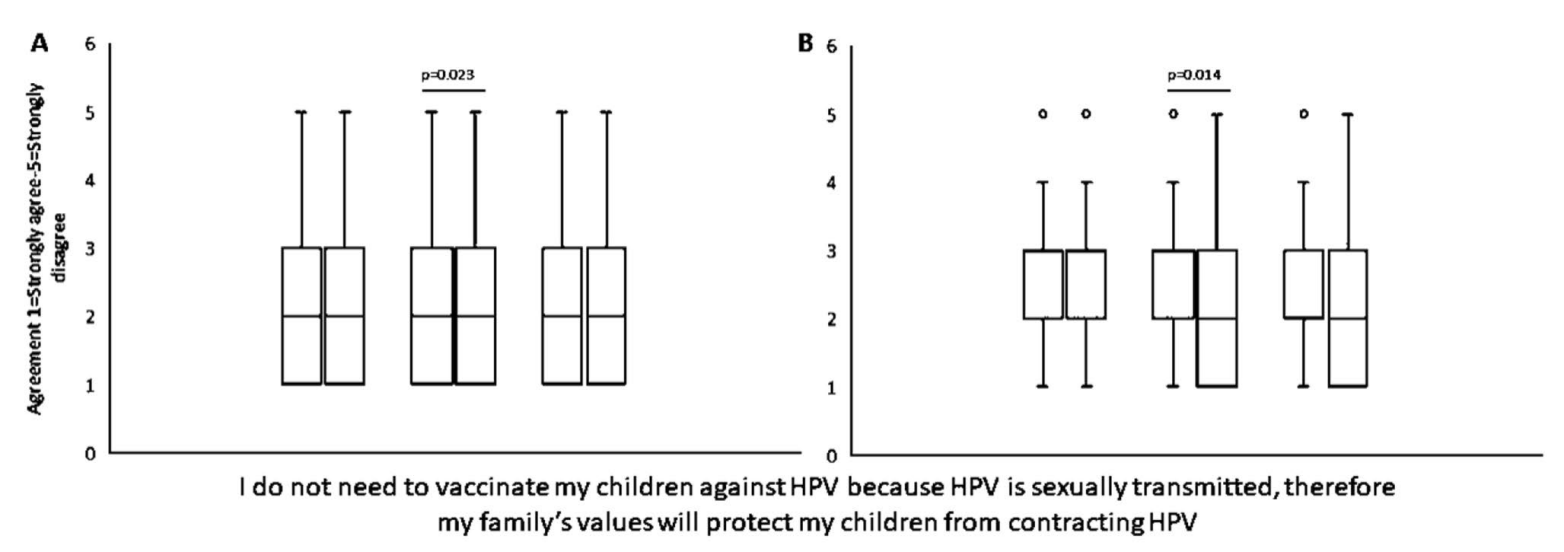
Change in belief about religious protection from HPV. Respondents were asked how much they agreed with the statement “I do not need to vaccinate my children against HPV because HPV is sexually transmitted, therefore my family’s values will protect my children from contracting HPV.” This question was asked prior to viewing the randomized intervention video, then again after. (**A**) Results show the median number of respondents who answered from 1 (strongly disagree) to 5 (strongly agree). Those who watched the religious video were significantly more likely to disagree with the statement (*p* = 0.023, effect size 0.13) as determined by Wilcoxon signed-rank test. The control video and the informational video did not significantly change the answers to this question. Control *n* = 433, Religious *n* = 394, and Informational *n* = 412. (**B**) Respondents were stratified according to their initial HPV vaccine intention score and children’s HPV vaccination status. Those deemed HPV vaccine hesitant were analyzed for changes in their responses to the statement. Those who watched the religious video had a significant (*p* = 0.0136, effect size 0.33) decrease in agreement with the statement that their values would protect them. Neither the control video (*p* = 0.266) nor the information video (*p* = 0.191) showed a significant change (N = Control 123; Religious 131; Informational 114)

To test the hypothesis that the religious video would decrease the feeling that family values protect against HPV and therefore vaccination is unnecessary, we asked the participants how much they agreed with the statement “I do not need to vaccinate my children against HPV because HPV is sexually transmitted, therefore my family’s values will protect my children from contracting HPV.” After watching the religious video, there was a significant change towards disagreement with this statement (*p* = 0.023, effect size 0.07), while those who watched the control or informational videos did not have a significant change in their response to this question (Fig. 5**).** When the HPV vaccine hesitant population was considered alone, the response to the video was even more evident. Those who watched the religious video had a significant decrease in their agreement with this statement (*p* = 0.014, effect size 0.33). Neither those who watched the control video (*p* = 0.27) or the informational video (*p* = 0.19) showed a significant change in the response to this item.

**Fig. 6:**
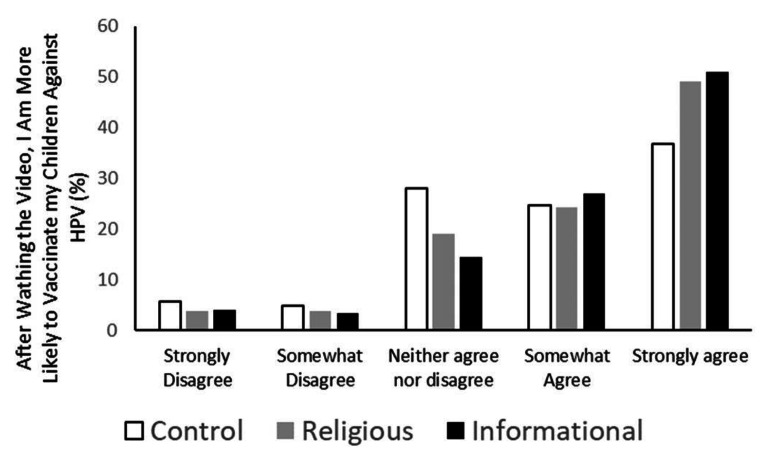
Self-reported influence of videos. Participants were asked how much they agreed with the statement “After watching the video, I am more likely to vaccinate my children.” The mean value of those who watched the religious and informational videos were each compared to those who watched the control video. Both the religious and informational videos were reported as having significantly more effect than the control (*p* < 0.001 for each) by Mann-Whitney U test. Control *n* = 433, Religious *n* = 394, and Informational *n* = 412

As an additional mechanism for measuring the utility of the intervention videos, one question in the survey asked, “Please indicate how much you agree with the following statement: After watching the video, I am more likely to vaccinate my children against HPV.” The religious intervention and the informational intervention were compared to the control intervention. Those who watched the religious or informational interventions reported significantly higher scores than those who watched the control videos (*p* < 0.001 for each, effect size 0.14, 0.17 respectively). (Fig. 6).

Knowledge of HPV disease was tested using four questions. These items primarily asked about the seriousness and prevalence of HPV infection. The questions were asked using a Likert scale with 1 being “Definitely true” and 5 being “Definitely false.” The questions were summed and the total was used to determine “knowledge of HPV infection.” Watching any of the three videos had a significant impact on knowledge of HPV infection (*p* value for each was less than 0.0001), The mean knowledge of HPV infection score was significantly higher after watching the religious video than the control (*p* < 0.0001) or the informational video than the control (*p* < 0.0001). There was no significant difference between those who watched the informational video and those who watched the religious video (*p* = 0.81).

Pearson correlation analysis and Chi-square analysis were used to determine if there were any correlations or associations between changes after viewing the videos and other characteristics, such as age, race, income level, sex, or religion. No significant associations were found. Overall vaccine attitudes were also examined before and after each video using the items “vaccines are more helpful than harmful,” “Vaccines often have severe side effects,” “vaccines contain dangerous toxins” and “Vaccines are effective at preventing disease” but no significant changes were found after viewing the videos (*p* > 0.05 for all).

## Discussion

The majority of the sample population viewed vaccines positively and indicated that they planned to vaccinate their children against HPV pre-intervention. Although this percentage seems high, (73%), it is still sub-optimal for HPV vaccine uptake, and the self-reports tend to be higher than actual HPV vaccination rates [14].

Wilcoxon signed-rank analysis showed that the religious intervention and the informational video improved intent to vaccinate against HPV, while the control intervention did not. The finding was statistically significant but the effect was small. The small effect was likely due to the presence in the sample of a large proportion of strongly pro-vaccine or strongly anti-vaccine individuals. Since the major focus of vaccine education efforts should be on those who can be affected by them, we subdivided the population and examined the “HPV vaccine hesitant” population. These individuals did not give either the highest or the lowest scores on the initial “HPV vaccine intention” measure, instead being found in the middle. They also indicated that their children were not fully vaccinated against HPV. These vaccine hesitant respondents made up 29.6% of the survey population. The effect of the video interventions was even more pronounced in the HPV vaccine hesitant individuals, with “moderate” effect sizes of 0.47 for the religious and 0.41 for the informational videos. These findings suggest that both interventions met their goals in influencing vaccine intention, especially among those who are capable of changing their minds or being influenced.

Alluvial analysis further showed that the intent to vaccinate among those who changed in attitude mainly increased from disagree or neutral to agree or strongly agree after viewing the video. Alluvial analysis also showed a similar increase in intent for participants who viewed the informational intervention. These findings indicate that both culturally-specific, storytelling approaches and informational approaches are likely to be effective.

The culturally-focused intervention had the power to influence a specific idea that is decreasing vaccination rates in this community: the idea that they are safe from HPV infection because of their values. This belief was significantly associated with lower intent to vaccinate in our previous work [23]. Viewing the religious-focused video significantly decreased this view in our population. The effect was especially strong in the HPV vaccine hesitant population, with an effect size of 0.33. Neither the control nor the informational video had a significant effect on responses to this item, indicating that the culturally-specific approach taken in this video is likely to be effective at influencing culturally-specific views.

Identifying the ideas or attitudes inhibiting vaccine acceptance, then making positive, culturally-relevant interventions that address them, is likely to be an effective strategy in improving vaccine attitudes. Considering that vaccine hesitant populations are often congregated in specific groups [31], an approach directed towards those groups is likely to be more effective than broad messaging.

A majority (70.7%) of participants stated that after watching the interventions they were more likely to vaccinate their children. There was a significantly greater influence of the religious and informational videos compared to the control, as measured by responses to the statement “After watching the video, I am more likely to vaccinate my children.” This shows the necessity of the control for this experiment, considering that even those watching the control video self-assessed as more likely to vaccinate after watching the video, even though to a lesser degree. It also suggests that both religious/cultural focused and informational video interventions are well-received and likely to be effective.

Our previous works found that hesitancy towards a specific vaccine is usually highly correlated with overall vaccine hesitancy [23, 32–35]. This remains true for HPV in this Christian population. However, the religious intervention video improved attitudes towards the HPV vaccine without significantly affecting overall vaccine hesitancy scores. This suggests that it is possible to decouple specific vaccines from overall hesitancy using targeted approaches.

Video-based messages have been found to be effective elsewhere. In Ghana, video interventions increased knowledge of HPV, cervical cancer, and translated to increased intent to vaccinate [36]. Other studies have also found value in culturally-focused approaches. Religiously-directed approaches to HPV vaccine advocacy showed significant results in a Christian population [28], and communicating to members through the church was found to increase trust in the vaccination message in a predominantly African-American Christian community [36]. The commonality in these studies is that video-based, culturally relevant messages are able to improve self-reported intent to vaccinate against HPV, and diminish some of the barriers to vaccination.

One limitation of the study is that we were only able to measure intent to vaccinate as reported by the respondents. Although many participants indicated that they intended to vaccinate their children and that the interventions made them view HPV vaccination more favorably, we were unable to measure if participants actually acted on their intent to vaccinate their children against HPV. Another limitation of the study is that the racial and ethnic makeup of the study is mostly white. This was likely due to the lack of racial selection filters in the sample. Specific interventions would likely be effective in resonating with members of individual ethnic groups. Similarly, this study only measures Christian Americans and the effectiveness with other groups may be different.

The number of participants in each group was slightly different. Although each group was selected randomly and evenly presented, this difference may have resulted from higher dropout for the religious video since it was slightly longer. Each video still had more than three times the number of participants needed according to the power analysis, but this may have introduced some unknown bias into the results.

## Conclusions

We conclude that educational interventions, whether informational or in the form of personal stories, can be effective at improving intent to vaccinate against HPV. We further conclude that a culturally-focused personal story can change specific attitudes that are detrimental to intent to vaccinate. These interventions were especially impactful in the HPV vaccine hesitant group, suggesting that, despite the difficulties in changing minds about vaccines, these types of approaches can be effective.

### Electronic supplementary material

Below is the link to the electronic supplementary material.

**Supplementary Material 1**: Supplemental Table S1

**Supplementary Material 2**: Supplemental Table S2
