# Supplementary material for: A Randomized Study to Determine the Effect of a Culturally Focused Video Intervention on Improving HPV Vaccine Intentions in a Christian Population in the United States
Source: J Community Health. 2024 Feb 23;49(4):661–73. doi: 10.1007/s10900-024-01327-8 (PMC11306300; doi:10.1007/s10900-024-01327-8)
Supplement: Supplementary file 2 — Supplementary Material 2: Supplemental Table S2 [file 10900_2024_1327_MOESM2_ESM.docx]

HPV Vaccine survey part 2

Start of Block: consent

Q1.1
Implied Consent
Title of the Research Study: HPV vaccine attitudes
 IRB ID#: IRB2022-194


My name is Brian Poole, PhD. I am a professor at Brigham Young University and I am conducting this research. You are being invited to participate in this research study about vaccination. I am interested to learn more about how you feel about the Human papillomavirus vaccine. Being in this study is optional. If you choose to be in the study, you will be asked to complete a survey, that should take approximately 15 minutes of your time. The survey will include a short video that you will watch, so please make sure you are in a place where you can listen to the sound. You can skip questions that you do not want to answer or stop the survey at any time. The survey is anonymous, and no one will be able to link your answers back to you. Please do not include your name or other information that could be used to identify you in the survey responses. You will receive your standard compensation from the survey provider for completing the survey. Questions? Please contact Brian Poole at brian_poole@byu.edu or 801-442-8092. If you have questions or concerns about your rights as a research participant, you can call the BYU Human Research Protection Program at 801-422-1461 or BYU.HRPP@byu.edu. If you want to participate in this study, click the Accept button to start the survey.

- Accept (1)
- Do not accept (2)

End of Block: consent

Start of Block: Block 7

Start of Block: demographics

Q2.1 We would like to gather a little bit of demographic data. These questions are helpful to researchers in comparing similar groups of people and to better understand survey results. Although these questions are of a personal nature, the researchers will not be able to connect the information with your name or anything else that could identify you. Please select the best answer for each question.

Q2.2 Religious affiliation

- Buddhism (1)
- Christianity (2)
- Hinduism (3)
- Islam (4)
- Judaism (5)
- Other (6)
- No religious affiliation (7)

Skip To: End of Survey If Religious affiliation != Christianity

| Page Break |  |
| --- | --- |

Q2.3 Do you have a child or children younger than the age of 11?

- Yes (1)
- No (2)

Skip To: End of Block If Do you have a child or children younger than the age of 11? = No

Q2.4 Number of children:

- 1 (1)
- 2 (2)
- More than 2 (3)

Q2.5 What is your age?

- Less than 18 (1)
- 18-25 (2)
- 26-35 (3)
- 36-45 (4)
- 46-55 (5)
- Over 55 (6)

Skip To: End of Block If What is your age? = Less than 18

Q2.6 Race: Please select all that apply:

- American Indian or Alaskan Native (1)
- Asian (2)
- Black or African American (3)
- Hispanic or Latino (4)
- Native Hawaiian or Pacific Islander (5)
- White (6)
- Prefer not to answer (7)
- Other (please specify) (8) __________________________________________________

| Page Break |  |
| --- | --- |

Q2.7 Sex

- Male (1)
- Female (2)
- Non-binary / third gender (3)
- Prefer not to say (4)

Q2.8 Martital status:

- Single (1)
- Partnered (2)
- Married (3)
- Divorced (4)
- Widow/widower (5)

| Page Break |  |
| --- | --- |

Q2.9 Education:

- Have not finished high school (1)
- Finished high school (2)
- Some college (3)
- Associate's degree (4)
- Bachelor's degree (5)
- Post-baccalaureate/professional degree (e.f. Masters, MD, DO, DDS, PhD, etc.) (6)

Q2.10 Political affiliation:

- Democrat (1)
- Republican (2)
- No political affiliation (3)
- I prefer not to answer (4)
- Other (please describe) (5) __________________________________________________

Q2.11 Please indicate your political leanings on economic issues

- Very liberal (1)
- Liberal (2)
- Somewhat liberal (3)
- Neither liberal nor conservative (4)
- Somewhat conservative (5)
- Conservative (6)
- Strongly conservative (7)

Q2.12 Please indicate your political leanings on scientific issues

- Very liberal (1)
- Liberal (2)
- Somewhat liberal (3)
- Neither liberal nor conservative (4)
- Somewhat conservative (5)
- Conservative (6)
- Strongly conservative (7)

Q2.13 Type of high school attended:

- Public (1)
- Private/Charter (2)
- Homeschooled (3)
- Other (please specify) (4) __________________________________________________

Q2.14 Type of school your children attend:

- Public (1)
- Private/Charter (2)
- Homeschool (3)
- A combination of the above (4)
- Other (please specify) (5) __________________________________________________

| Page Break |  |
| --- | --- |

Q2.15 How many people (not including yourself) live in your home?

- 0 (1)
- 1 (2)
- 2 (3)
- 3 (4)
- More than 3 (5)

Q2.16 What is your current employment status? Check **ALL** that apply.

- Working full time for pay (Input number of hours below) (1) __________________________________________________
- Working part time for pay (Input number of hours below) (2) __________________________________________________
- Not currently employed, looking for work (3)
- Retired (4)
- Stay at home parent (5)
- Disabled (not working because of permanent or temporary disability) (6)
- Other (please specify) (7) __________________________________________________

Q2.17 Which category best describes your yearly household income before taxes? Include all income received from employment, social security, bank interest, retirement accounts, rental property, investments, etc.

- Less than $5,000 (1)
- $5,000-$9,999 (2)
- $10,000-$14,999 (3)
- $15,000-$19,999 (4)
- $20,000-$29,999 (5)
- $30,000-$39,999 (6)
- $40,000-$49,999 (7)
- $50,000-$59,999 (8)
- $60,000-$74,999 (9)
- $75,000-$99,999 (10)
- $100,000-$124,999 (11)
- $125,000-$149,999 (12)
- $150,000 or more (13)

| Page Break |  |
| --- | --- |

Q2.18 Specific Religious Affiliation (Christianity):

- Anglican/Episcopalian (1)
- Baptist (2)
- Catholic (3)
- Christian (non-denominational) (4)
- Church of Christ/Disciples of Christ (5)
- Congregational (6)
- Jehovah's Witness (7)
- LDS (Mormon) (8)
- Lutheran (9)
- Methodist/Wesleyan (10)
- Orthodox (Eastern) (11)
- Pentecostal/Charismatic (12)
- Protestant (Other) (13)
- Reformed/Presbyterian (14)
- Seventh-day Adventist (15)
- Other (please specify) (16) __________________________________________________

End of Block: demographics

Start of Block: HPV pre-video survey

Q3.1 Please rate how much you agree with the following statements about Human Papilloma Virus (HPV) and sexual behavior. HPV is transmitted sexually, and can cause certain types of cancer. Vaccination to prevent HPV is often offered for children in the pre-teen years.

|  | Strongly disagree (1) | Somewhat disagree (2) | Neither agree nor disagree (3) | Somewhat agree (4) | Strongly agree (5) |
| --- | --- | --- | --- | --- | --- |
| I do not need to vaccinate my children against HPV because HPV is sexually transmitted, therefore my family's values will protect my children from contracting HPV. (1) |  |  |  |  |  |
| The fear of getting HPV helps prevent premarital sex. (2) |  |  |  |  |  |
| People with diseases caused by HPV are responsible for their own suffering, because the virus is only transmitted through promiscuous sexual practices. (3) |  |  |  |  |  |
| Vaccinating my children against HPV sends them mixed messages about sexual activity. (4) |  |  |  |  |  |
| Vaccinating my children against HPV will make them more likely to have premarital sex. (5) |  |  |  |  |  |

| Page Break |  |
| --- | --- |

Q3.2 We would now like to ask you a few questions about the Human Papillomavirus Vaccine. Please rate how much you agree with the following statements about the Human Papillomavirus (HPV) vaccine. HPV is a primarily sexually transmitted virus. Vaccination for HPV is often offered for pre-teens.

|  | Strongly disagree (1) | Somewhat disagree (2) | Neither agree nor disagree (3) | Somewhat agree (4) | Strongly agree (5) |
| --- | --- | --- | --- | --- | --- |
| I am likely to vaccinate my children against HPV OR I have already vaccinated my eligible children against HPV. (1) |  |  |  |  |  |
| I am likely to recommend that others vaccinate their children against HPV. (2) |  |  |  |  |  |
| The potential side effects of the HPV vaccine will prevent me from vaccinating my children against HPV. (3) |  |  |  |  |  |
| Since HPV is sexually transmitted, I will not vaccinate my children against it. (4) |  |  |  |  |  |
| I will (or already have) vaccinated both my daughters and sons against HPV. (5) |  |  |  |  |  |
| The HPV vaccine would protect my child in the case of sexual assault. (6) |  |  |  |  |  |

| Page Break |  |
| --- | --- |

Q3.3 Please rate how much you agree with the following statements about vaccines in general

|  | Strongly disagree (1) | Somewhat disagree (2) | Neither agree nor disagree (3) | Somewhat agree (4) | Strongly agree (5) |
| --- | --- | --- | --- | --- | --- |
| Vaccines are more helpful than harmful. (1) |  |  |  |  |  |
| Vaccines often have severe side effects. (2) |  |  |  |  |  |
| Vaccines contain dangerous toxins. (3) |  |  |  |  |  |
| Vaccines are effective at preventing disease. (4) |  |  |  |  |  |

| Page Break |  |
| --- | --- |

Q3.4 Now we would like to ask you a few questions about HPV infection and disease. Please rate the following statements about HPV

|  | Definitely true (1) | Probably true (2) | Neither true nor false (3) | Probably false (4) | Definitely false (5) |
| --- | --- | --- | --- | --- | --- |
| HPV is a potentially life-threatening infection. (1) |  |  |  |  |  |
| HPV infection can cause severe physical suffering. (2) |  |  |  |  |  |
| Only a small minority of people will catch HPV during their lives. (3) |  |  |  |  |  |
| HPV causes a substantial amount of cancer. (4) |  |  |  |  |  |
| HPV causes cancer in women but not men. (5) |  |  |  |  |  |
| The HPV vaccine is effective at preventing almost all cancers caused by HPV. (6) |  |  |  |  |  |

| Page Break |  |
| --- | --- |

Q3.5 Please rate the truth of the following statements about vaccines in general.

|  | Definitely true (1) | Probably true (2) | Neither true nor false (3) | Probably false (4) | Definitely false (5) |
| --- | --- | --- | --- | --- | --- |
| Smallpox has been eliminated because of mass vaccination. (1) |  |  |  |  |  |
| Vaccination increase the risk of allergies. (2) |  |  |  |  |  |
| Unvaccinated children are more resistant to infections. (3) |  |  |  |  |  |
| Routine childhood vaccines can be given to a child taking antibiotics for an ear infection . (4) |  |  |  |  |  |
| Current scientific evidence supports associations between vaccines and chronic conditions such as autism or multiple sclerosis. (5) |  |  |  |  |  |
| The Food and Drug Administration (FDA) approval process for vaccines is the same as that for other drugs and pharmaceuticals. (6) |  |  |  |  |  |

Q3.6 These questions will ask about your religious activity and attendance at religious functions. Please answer how often you did these things WITHOUT COVID restrictions: for example, before restrictions on attending church in person were in place.

|  | Less than once a month (1) | More than once a month (2) | Once a week (3) | More than once a week (4) | Once a day (5) | More than once a day (6) |
| --- | --- | --- | --- | --- | --- | --- |
| How often do you read scriptures/holy texts? (1) |  |  |  |  |  |  |
| How often do you attend Sunday School, religious classes or seminars? (2) |  |  |  |  |  |  |
| How often do you pray? (3) |  |  |  |  |  |  |
| How often do you attend organized worship services? (4) |  |  |  |  |  |  |
| How often do you attend other activities sponsored by a religious group? (5) |  |  |  |  |  |  |

| Page Break |  |
| --- | --- |

Q3.7 Please answer the following questions about the influence of your religion on your life.

|  | No influence (1) | Minimal influence (2) | Some influence (3) | Moderate influence (4) | Strong influence (5) | Extreme influence (6) |
| --- | --- | --- | --- | --- | --- | --- |
| How much influence do your religious beliefs have on what you wear? (1) |  |  |  |  |  |  |
| How much influence do your religious beliefs have on what you eat and drink? (2) |  |  |  |  |  |  |
| How much influence do your religious beliefs have on your choices about whom you associate with? (3) |  |  |  |  |  |  |
| How much influence do your religious beliefs have on what social activities you undertake? (4) |  |  |  |  |  |  |
| To what extent do your religious beliefs impact the important decisions that you make? (5) |  |  |  |  |  |  |

| Page Break |  |
| --- | --- |

Q3.8 Please rate how much you agree with the following statements about religious hope.

|  | Strongly disagree (1) | Somewhat disagree (2) | Neither agree nor disagree (3) | Somewhat agree (4) | Strongly agree (5) |
| --- | --- | --- | --- | --- | --- |
| A positive afterlife/Heaven exists. (1) |  |  |  |  |  |
| It is possible for all humans to live in harmony together. (2) |  |  |  |  |  |
| Miracles are real. (3) |  |  |  |  |  |
| My suffering will be rewarded. (4) |  |  |  |  |  |
| In the future, my children will be able to lead a better life. (5) |  |  |  |  |  |

| Page Break |  |
| --- | --- |

Q3.9 Please indicate how much you agree with the following statement: My religion promotes the use of vaccines.

- Strongly disagree (1)
- Somewhat disagree (2)
- Neither agree nor disagree (3)
- Somewhat agree (4)
- Strongly agree (5)

Q3.10 Please indicate how much you agree with the following statement: People who share my religion vaccinate their children.

- Strongly disagree (1)
- Somewhat disagree (2)
- Neither agree nor disagree (3)
- Somewhat agree (4)
- Strongly agree (5)

Q3.11 Which do you feel your religion would most **disagree** with:

- Everyone should be vaccinated (1)
- People should make up their own minds about how and when to vaccinate themselves and/or their children, but vaccines are a good thing (2)
- The religion does not teach one way or the other about vaccines (3)
- People should make up their own minds about how and when to vaccinate themselves and/or their children, but vaccines are not encouraged (4)
- Vaccines should not be used (5)

| Page Break |  |
| --- | --- |

Q3.12 How strongly do you agree with the following statement: My religion encourages me not to engage in sexual behaviors unless I am married to the other individual.

- Strongly disagree (1)
- Somewhat disagree (2)
- Neither agree nor disagree (3)
- Somewhat agree (4)
- Strongly agree (5)

Q3.13 Rate how much you agree with the following statement: Having sex before marriage is a sin.

- Strongly disagree (1)
- Somewhat disagree (2)
- Neither agree nor disagree (3)
- Somewhat agree (4)
- Strongly agree (5)

Q3.14 How much emphasis does your religion place on sexual behavior?

- One of the least emphasized parts (1)
- Less emphasized than most other parts (2)
- Equally emphasized as other parts (3)
- More emphasized than most other parts (4)
- One of the most emphasized parts (5)

| Page Break |  |
| --- | --- |

Q3.15 How much did your parents or caretakers emphasize certain rules or cautions about sexual behavior (such as abstinence before marriage or in a committed relationship) in what they taught you?

- Never (1)
- Almost never (2)
- Sometimes taught but not emphasized (3)
- A lot (4)
- Excessively (5)

Q3.16 To what extent are sexual relationships outside of marriage discouraged within your social group?

- Not discouraged at all (1)
- Rarely discouraged (2)
- Somewhat discouraged (3)
- Discouraged (4)
- Strongly discouraged (5)
- Extremely discouraged (6)

Q3.17 As a parent, I plan to teach about sexual behavior outside of marriage as follows:

- Not discouraged at all (1)
- Rarely discouraged (2)
- Somewhat discouraged (3)
- Discouraged (4)
- Strongly discouraged (5)
- Extremely discouraged (6)

| Page Break |  |
| --- | --- |

Q3.18 We'd like to ask you about your opinions about modern medicine. Please rate how much you agree with the following statements about Modern Medicine

|  | Strongly disagree (1) | Somewhat disagree (2) | Neither agree nor disagree (3) | Somewhat agree (4) | Strongly agree (5) |
| --- | --- | --- | --- | --- | --- |
| Doctors (in general) care about their patients' health just as much or more than their patients do. (1) |  |  |  |  |  |
| Doctors usually pay attention to and have regard for what their patients are telling them. (2) |  |  |  |  |  |
| Doctors are competent, careful and well trained. (3) |  |  |  |  |  |
| Doctors are totally honest in telling their patients about all of the different treatment options available for their conditions. (4) |  |  |  |  |  |
| Prescribed treatments are more beneficial than harmful. (5) |  |  |  |  |  |
| A doctor would never mislead you about anything. (6) |  |  |  |  |  |
| Doctors think only about what is best for their patients. (7) |  |  |  |  |  |
| Doctors always use their very best skill and effort on behalf of their patients. (8) |  |  |  |  |  |

| Page Break |  |
| --- | --- |

Q3.19 Have you received all your recommended vaccines?

- I have not received any vaccines (1)
- I only have my newborn vaccinations (2)
- I have received all my vaccinations into adolescence, but none after (3)
- I have received some vaccinations during adulthood (4)
- I have received all the vaccinations that have been recommended to me (5)

Q3.20 Have you/do you plan to make sure your children all received their recommended vaccinations?

- I do not have children (1)
- My children have received no vaccines (2)
- My children have received only their newborn vaccinations (3)
- My children have received some childhood vaccines, but not all (4)
- My children have received all recommended vaccines for their age group (5)
- I am unsure whether my children have received all the vaccinations recommended for their age group (6)

Q3.21 The HPV vaccine is given in several doses. Please indicate how complete your HPV vaccination is.

- I have not received any vaccines for HPV (1)
- I have received some of the vaccines for HPV (2)
- I have received all the vaccines for HPV (3)

Q3.22 The HPV vaccine is given in several doses. Please indicate how complete your children’s HPV vaccinations are.

- My children have not received any vaccines for HPV (1)
- My children have all received some of the vaccines for HPV (2)
- My children have received all the vaccines for HPV (3)

End of Block: HPV pre-video survey

Start of Block: Please watch the following video.

Q4.1 Please watch the following video. You will be able to proceed to the remainder of the survey after the video.

Q4.2

Q4.3 Timing

First Click (1)

Last Click (2)

Page Submit (3)

Click Count (4)

End of Block: Please watch the following video.

Start of Block: Please watch the following video

Q5.1 Please watch the following video. You will be able to proceed to the remainder of the survey after the video.

Q5.2

Q5.3 Timing

First Click (1)

Last Click (2)

Page Submit (3)

Click Count (4)

End of Block: Please watch the following video

Start of Block: Please watch the following video

Q6.1 Please watch the following video. You will be able to proceed to the remainder of the survey after the video.

Q6.2

Q6.3 Timing

First Click (1)

Last Click (2)

Page Submit (3)

Click Count (4)

End of Block: Please watch the following video

Start of Block: HPV post-video survey

Q7.1 Please answer the following questions based on your thoughts after watching the provided video about HPV.

Q7.2 Please rate how much you agree with the following statements about Human Papilloma Virus (HPV) and sexual behavior. HPV is transmitted sexually, and can cause certain types of cancer. Vaccination to prevent HPV is often offered for children in the pre-teen years.

|  | Strongly disagree (1) | Somewhat disagree (2) | Neither agree nor disagree (3) | Somewhat agree (4) | Strongly agree (5) |
| --- | --- | --- | --- | --- | --- |
| I do not need to vaccinate my children against HPV because HPV is sexually transmitted, therefore my family's values will protect my children from contracting HPV. (1) |  |  |  |  |  |
| The fear of getting HPV helps prevent premarital sex. (2) |  |  |  |  |  |
| People with diseases caused by HPV are responsible for their own suffering, because the virus is only transmitted through promiscuous sexual practices. (3) |  |  |  |  |  |
| Vaccinating my children against HPV sends them mixed messages about sexual activity. (4) |  |  |  |  |  |
| Vaccinating my children against HPV will make them more likely to have premarital sex. (5) |  |  |  |  |  |

| Page Break |  |
| --- | --- |

Q7.3 We would now like to ask you a few questions about the Human Papillomavirus Vaccine. Please rate how much you agree with the following statements about the Human Papillomavirus (HPV) vaccine. HPV is a primarily sexually transmitted virus. Vaccination for HPV is often offered for pre-teens.

|  | Strongly disagree (1) | Somewhat disagree (2) | Neither agree nor disagree (3) | Somewhat agree (4) | Strongly agree (5) |
| --- | --- | --- | --- | --- | --- |
| I am likely to vaccinate my children against HPV OR I have already vaccinated my eligible children against HPV. (1) |  |  |  |  |  |
| I am likely to recommend that others vaccinate their children against HPV. (2) |  |  |  |  |  |
| The potential side effects of the HPV vaccine will prevent me from vaccinating my children against HPV. (3) |  |  |  |  |  |
| Since HPV is sexually transmitted, I will not vaccinate my children against it. (4) |  |  |  |  |  |
| I will (or already have) vaccinated both my daughters and sons against HPV. (5) |  |  |  |  |  |
| The HPV vaccine would protect my child in the case of sexual assault. (6) |  |  |  |  |  |

| Page Break |  |
| --- | --- |

Q7.4 Please rate how much you agree with the following statements about vaccines in general

|  | Strongly disagree (1) | Somewhat disagree (2) | Neither agree nor disagree (3) | Somewhat agree (4) | Strongly agree (5) |
| --- | --- | --- | --- | --- | --- |
| Vaccines are more helpful than harmful. (1) |  |  |  |  |  |
| Vaccines often have severe side effects. (2) |  |  |  |  |  |
| Vaccines contain dangerous toxins. (3) |  |  |  |  |  |
| Vaccines are effective at preventing disease. (4) |  |  |  |  |  |

| Page Break |  |
| --- | --- |

Q7.5 Now we would like to ask you a few questions about HPV infection and disease. Please rate the following statements about HPV

|  | Definitely true (1) | Probably true (2) | Neither true nor false (3) | Probably false (4) | Definitely false (5) |
| --- | --- | --- | --- | --- | --- |
| HPV is a potentially life-threatening infection. (1) |  |  |  |  |  |
| HPV infection can cause severe physical suffering. (2) |  |  |  |  |  |
| Only a small minority of people will catch HPV during their lives. (3) |  |  |  |  |  |
| HPV causes a substantial amount of cancer. (4) |  |  |  |  |  |
| HPV causes cancer in women but not men. (5) |  |  |  |  |  |
| The HPV vaccine is effective at preventing almost all cancers caused by HPV. (6) |  |  |  |  |  |

| Page Break |  |
| --- | --- |

Q7.6 Please indicate how much you agree with the following statement: My religion promotes the use of vaccines.

- Strongly disagree (1)
- Somewhat disagree (2)
- Neither agree nor disagree (3)
- Somewhat agree (4)
- Strongly agree (5)

Q7.7 Please indicate how much you agree with the following statement: People who share my religion vaccinate their children.

- Strongly disagree (1)
- Somewhat disagree (2)
- Neither agree nor disagree (3)
- Somewhat agree (4)
- Strongly agree (5)

| Page Break |  |
| --- | --- |

Q7.8 Please indicate how much you agree with the following statement: After watching the video, I am more likely to vaccinate my children against HPV.

- Strongly disagree (1)
- Somewhat disagree (2)
- Neither agree nor disagree (3)
- Somewhat agree (4)
- Strongly agree (5)

Q7.9 Please explain your answer to the previous question.

________________________________________________________________

End of Block: HPV post-video survey
